# Socio-Economic Gradients in Maternal and Child Health-Seeking Behaviours in Egypt: Systematic Literature Review and Evidence Synthesis

**DOI:** 10.1371/journal.pone.0093032

**Published:** 2014-03-24

**Authors:** Lenka Benova, Oona M. R. Campbell, George B. Ploubidis

**Affiliations:** 1 Department of Population Health, Faculty of Epidemiology and Population Health, London School of Hygiene and Tropical Medicine, London, United Kingdom; 2 Department of Infectious Disease Epidemiology, Faculty of Epidemiology and Population Health, London School of Hygiene and Tropical Medicine, London, United Kingdom; Aga Khan University, Pakistan

## Abstract

**Background:**

Health-seeking behaviour lies on the direct pathway between socio-economic position (SEP) and health outcomes. The objective of this systematic review is to identify and synthesise evidence of socio-economic gradients in health-seeking behaviours related to maternal and child health in Egypt.

**Methods:**

Four databases (Medline, Embase, Global Health and Web of Science) were searched in September 2013 for material published in English from 1992 to 2013 for a combination of terms describing health-seeking behaviours, indicators of socio-economic position and geographical limitation to Egypt. Findings of studies were described and synthesised in a narrative format as meta-analysis was not possible.

**Findings:**

Among the 786 references identified, 10 articles met the inclusion criteria. Six studies examined maternal and five studies child health-seeking behaviours (one study examined both). For maternal health, three dimensions of health-seeking behaviour (receipt of any care, type of care and intensity of care) were covered by studies of ante-natal and one dimension (type of care) by analyses of delivery care. For child health, two dimensions of preventive care (coverage of and intensity of immunisation) and three dimensions of curative care (receipt of any care, type and cost of care) were analysed.

**Conclusions:**

Based on two studies of time trends in nationally-representative surveys, socio-economic inequalities in seeking care for basic preventive and curative interventions in maternal and child health appear to have narrowed. Limited evidence of gradients in intensity of maternal preventive and provider selection in child curative care showed that inequalities may have widened. In studies of more geographically and socially homogeneous samples, fewer gradients were identified. Current body of evidence contains numerous limitations and gaps and is insufficient to draw a conclusive summary of such gradients. Improved understanding of SEP gradients is crucial in designing and prioritising interventions to equitably improve maternal and child health outcomes.

## Introduction

In virtually every context where they have been studied, inequalities in health outcomes based on living standards or on social hierarchy (as observed through various measures of socio-economic position, SEP) have been identified.[1] This association is hypothesised to arise mainly on the basis of causal pathways, encompassing healthcare access and utilisation, psychosocial determinants, health knowledge and behaviours, as well as environmental hazards.[2]–[5] As one of these pathways, health-seeking behaviour comprises several sequential decisions and actions through which individuals proceed in their contact with the healthcare system; including experiencing and reporting symptoms, seeking care, choosing a provider, paying for care, adhering to treatment, as well as timing and intensity of follow-up visits. Health-seeking behaviour includes preventive (immunisation or screening) and curative care (after the onset of symptoms).

A review of socio-economic determinants of health-seeking behaviour in low and middle income countries indicated that a variety of individual and households-level indicators have been used to reflect socio-economic position, including education, occupation, absolute or relative poverty level, and access to material, financial and productive resources (income, landholding, assets).[6] Rigorously evaluated interventions, such as conditional cash transfer (CCT) programs, showed that provision of cash transfers to female heads of households can lead to an increase in healthcare utilization patterns.[7] Socio-economic status is hypothesised to influence health-seeking behaviour through several mechanisms, such as material and intellectual resources and access to health facilities.[8], [9] However, individuals rarely make health-related decisions in a social vacuum and their socio-economic position is not solely an individual-level characteristic. Therefore, the association between SEP and health-seeking behaviour can be examined on various levels, spanning from the individual and familial environment, to the wider community and country.

### Objectives

The objective of this systematic review is to identify and synthesise evidence published in the previous two decades about the existence, magnitude and trends in socio-economic gradients in health-seeking behaviours related to maternal and child health in Egypt. Firstly, a summary of the types and dimensions of health-seeking behaviour analysed by included studies is presented. Secondly, we synthesise the evidence regarding the extent of gradients identified, and lastly, limitations of current evidence and recommendations for future research are outlined. Maternal health-seeking behaviour for the purposes of this review refers to the timing and intensity of care, as well as provider choice and cost incurred during pregnancy, childbirth and in the immediate post-partum period. Child health-seeking behaviour is defined as actions taken in relation to healthcare for children under five years of age. Preventive and curative health-seeking behaviours are included.

## Methods

### Data sources and search strategy

Four databases (Medline, Embase, Global Health and Web of Science) were searched in September 2013 for material published in English from 1992 to 2013. Where available, MeSH terms were combined with free-text terms capturing components of health-seeking behaviour (access, utilization, provider selection, and cost of care), a wide range of indicators of socio-economic position (education, literacy, employment, wealth, income, consumption, expenditure, assets, poverty, indebtedness) and geographic limitation to Egypt. The reference lists of included articles were also screened. The complete search strategy is presented in Table 1. The review is reported according to the Preferred Reporting Items for Systematic Reviews and Meta-Analyses (PRISMA) guideline (Table S1).[10] The review protocol was not registered.

**Table 1:** MeSH and text search terms used in databases searched according to algorithm (1 AND 2 AND 3).

Search Concept	Text search terms	MeSH terms
**Database**	(Medline, Embase, Global Health and Web of Science)	(Medline, Embase and Global Health)
**1. Health-seeking behaviour**	((health OR health-care OR healthcare OR health-related OR provider OR help OR care OR therap* OR treatment) AND (seeking OR behavio?r OR decision OR choice* OR utili?ation OR narrative OR network)) OR ((illness OR sick*) AND (perception OR narrative)) OR ((out?of?pocket OR private OR health) AND (expen* OR cost* OR payment OR fee OR charge)) OR pathways to doctor OR pathways to the doctor OR health-seeking OR help-seeking OR care-seeking	**Medline:** Attitude to Health, Health Behavior, Illness Behavior, Direct Service Costs, Cost of Illness, Fees, Medical. **Embase:** health related behavior, patient attitude, health care utilization, health care access. **Global Health:** health care utilization, social barriers, Health care costs.
**2. Socio-economic determinants**	socio-economic OR socioeconomic OR social status OR social class OR social position OR economic position OR poverty OR inequalit* OR gradient* OR deprivation OR SES OR SEP OR employment OR occupation OR unemployment OR education* OR school* OR graduat* OR literacy OR numeracy OR income OR wage OR pension OR salary OR wealth OR asset* OR loan OR debt OR borrow* OR consumption OR expenditure OR spend* OR housing OR crowding OR determinant* OR sociodemographic	**Medline:** Socioeconomic Factors, Sociometric Techniques, Social Class. **Embase:** Social status. **Global Health:** Socioeconomic status.
**3. Egypt**	Egypt*	**Medline, Embase, Global Health:** Egypt.

* Truncation symbol.

### Study selection and data extraction

Abstracts of all references identified in searches were screened and discarded if they did not contain data from Egypt or maternal or child health-seeking behaviour as an outcome measure. Cognizant of the fact that SEP indicators may be included in analyses as confounders and may thus not be identified as exposures in the abstracts, full-text versions of remaining references were screened. At this stage, we eliminated studies without SEP as an exposure or confounder, those in which the SEP variable used was not clearly defined, and those not presenting any statistical test (p value, confidence interval or standard error) of the association between SEP and health-seeking behaviour. Descriptive information abstracted from studies included study design, study objective, study population and data sources, type of health-seeking behaviour examined and measures of SEP used. We classified each study according to its main objective into one of the following three groups: SEP was the main determinant examined by the study, SEP was one of the several risk factors examined by the study or SEP was one of the confounders used in the study. Abstracted information on study findings included type of statistical analysis and tests used, crude and adjusted parameters that quantified the association between SEP measure(s) and health-seeking behaviour outcomes and confounders included in adjusted analyses, if any. No data were obtained from investigators, but results were confirmed with authors of Stephenson et al (2012). For the results of Gwatkin et al (2007), binary significance levels of the concentration indices (<0.05, >0.05) were calculated by using the standard errors by this study.

### Analysis

No meta-analysis was performed due to insufficient number of comparable estimates for any single health-seeking behaviour. Findings of studies were described and synthesised in a narrative format. The results of analyses are presented separately for each dimension of health-seeking behaviour: 1) Seeking any care, 2) Type of care sought/received, 3) Intensity of care received, and 4) Cost of care incurred, based on a conceptual approach used by Pokhrel and colleagues.[11] A risk of bias assessment was developed based on the Newcastle-Ottawa quality assessment scale.[12] This tool was used in consideration of the strengths and limitations of the available evidence.

## Results

**Figure 1:**
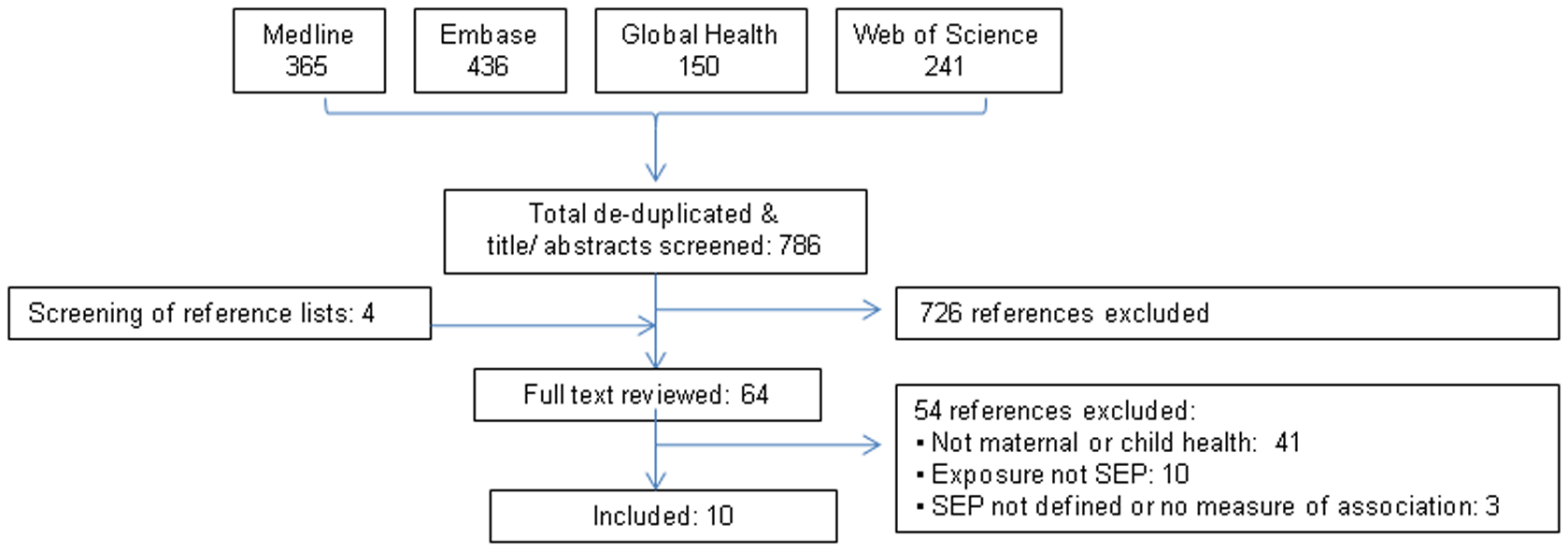
Systematic review search and inclusion flowchart.

A total of 786 unique references were identified and screened. The majority (83%) of excluded references did not examine any component of health-seeking behaviour. From 64 references reviewed in full-text, 10 articles met the inclusion criteria (Figure 1). Six studies examined maternal health-seeking behaviours and five studies child health-seeking behaviours (Tables 2 and 3, respectively, studies listed in chronological order by year of publication); one study included both behaviours. Among the ten included studies, nine set out to examine the association between socio-economic position and health-seeking behaviour, either as the main exposure or one of several main exposures.

**Table 2:** Descriptive characteristics of included maternal health-seeking behaviour studies.

#	Author, Year	Study design	Study sample and objective	Exposure: SEP definition	Outcome: Health-seeking behaviour(s) – type, definition and level in sample
1	Abdel Houssein, 1997[16]	Cross-sectional	Pregnant women in third trimester attending 6 randomly selected MCH centers in Alexandria, all pregnant women in third trimester interviewed (no date of study provided), n = 240. SEP: One of risk factors.	**IS: Education level** (no information on number or types of categories). **HH: Family SES score** based on education (woman and husband), monthly per capita income (no further information), crowding and household sanitation.	**Pattern of attendance of ANC clinics:** based on timing of initial visit (1^st^ ANC visit during 1^st^ trimester) and total number of ANC visits for current pregnancy; classified (no further information) into adequate and inadequate. Level: 28.8% adequate.
2	Yassin et al., 2003[17]	Cross-sectional	6 villages in Beni Sueif, all mothers of children less than one year old living in an area around a sentinel site (no date of study provided), n = 231. SEP: One of risk factors.	**IS: Literacy** (binary), **occupation** (binary). **IO: Spouse literacy** (binary), **spouse occupation** (binary). **HH: Land ownership** (binary), **house ownership** (binary), **cattle ownership** (binary), **access to potable water** (binary), **meat consumed <4 times in previous month** (binary).	**Receipt of any ANC** at local primary center during most recent pregnancy (with child <1 year old). Level: 33.8% received any ANC.
3	Gwatkin et al., 2007[13]	Cross-sectional	EDHS 1995, 2000. Population representative sample of ever-married women. Last birth among women with at least one birth in the five years preceding the survey, n: 1995 = 7797, 2000 = 7953. SEP: Main determinant.	**HH: DHS Wealth index quintile.**	**Any ANC by a medically-trained person** (at least one consultation from a doctor, nurse or trained midwife). Level: 1995: 42.5%, 2000: 55.7%. **Multiple (3+) ANC visits with medically-trained person.** Level: 1995: 34.9%, 2000: 43.7%. **Delivery with skilled attendance** (doctor, nurse or midwife). Level: 1995: 46.2%, 2000: 60.9%. **Delivery location: Public facility.** Level: 1995: 17.9%, 2000: 22.2%. **Delivery location: Private facility.** Level: 1995: 14.6%, 2000: 26.0%. **Delivery location: Home (woman’s or another home).** Level: 1995: 67.0%, 2000: 51.7%.
4	Khadr, 2009[14]	Cross-sectional	EDHS 1995, 2000, 2005 Population representative sample of ever-married women. Last birth among currently married women with at least one birth in the 5-years preceding survey; n: 1995 = 7828, 2000 = 7823, 2005 = 9744. SEP: Main determinant.	**IS: Education level** (no education, some primary, primary to secondary, completed secondary and higher). **HH: DHS Wealth index quintile.**	**Any ANC received** (no further definition). Level: 1995: 42.4%, 2000: 54.1%, 2005: 71.4%. **Regular ANC received** (4+ visits during pregnancy). Level: 1995: 31.0%, 2000: 40.7%, 2005: 61.3%. **Delivery with skilled attendance** (no further definition). Level: 1995: 41.7%, 2000: 55.8%, 2005: 70.5%. **Home delivery.** Level: 1995: 64.5%, 2000: 49.1%, 2005: 33.6%.
5	Chiang et al., 2012[18]	Cross-sectional	2007 survey of married women <50 years from a Giza village, n = 189. SEP: One of risk factors.	**IS: Woman's education** (no primary, completed primary or higher), **existence of cash income** (binary). **IO: Husband's education** (primary school or lower, higher than primary).	**Regular ANC (4+ visits) during most recent pregnancy. Level: 48.7%. Delivery with skilled medical professional at most recent delivery.** Level: 79.4%. **Delivery in a health facility at most recent delivery.** Level: 61.4%.
6	Stephenson et al., 2012[15]	Cross-sectional	EDHS 2008. Population representative sample of ever-married women. Last birth among women with at least one birth in the five years preceding the survey, n = 7813. SEP: One of risk factors.	**C: Community-level economic prosperity** - mean score of household wealth (DHS wealth score) per PSU (primary sampling unit), continuous variable.	**Any ANC received** (from a medically trained person). Level: 73.23%. **Regular ANC received** (4+ visits). Level: 65.75%. **First ANC visit received in 1^st^ trimester.** Level: 59.48%. **Delivery in a health facility.** Level: 71.25%.

EDHS – Egypt Demographic and Health Survey. Measures of SEP: **IS**: Individual – self, **IO:** Individual– other (spouse, mother, etc), **HH:** Household-level, **C**: Community-level.

**Table 3:** Descriptive characteristics of included child health-seeking behaviour studies.

#	Author, Year	Study design	Study sample and objective	Exposure: SEP – type, definition, level in sample	Outcome: Health-seeking behaviour(s) – type, definition and level in sample
1	Reichler et al., 1998[26]	Cross-sectional	Nationally-representative survey of children under 48 months of age surveyed one month after second National Immunisation Day (NID, polio) round in 1995, n = 4188 children from 3216 households. SEP: One of risk factors.	**HH: Radio in household** (binary), **TV in household** (binary).	**Number of doses of OPV (oral poliomyelitis vaccine) received.** Level: 0 doses: 9% (CI95%: 7.1%–9.9%). Level: 1 dose: 17% (CI95%: 14.6%–19.7%). Level: 2 doses: 74% (CI95%: 71.4%–77.3%).
2	Yount, 2003[27]	Cross-sectional.	Two Governorates Linkages Survey 1995–1997, children <5 years with diarrhoea in past 2 weeks in 12 rural villages of Minya governorate, n = 129 children, 152 episodes of care-seeking. SEP: Confounder in analysis of gender.	**IO: Maternal education** (binary). **HH: Major asset ownership** (binary).	If reported symptoms of diarrhoea: **Type of provider sought** (episodes, n = 152). Other:15%, Lay:16%, Pharmacist: 40%, Doctor: 29%. **Location of care** (episodes, n = 152). Other: 28%, Public:12%, Private: 21%, Pharmacy: 39%. **Cost of care** (children, n = 129). Free, ≤ 1 EPG, > 1 EGP (Proportions by category not provided, but median expenditure for boys 0.5 EGP, girls 0.2 EPG).
3	Yount, 2004[28]	Cross-sectional	Two Governorates Linkages Survey 1995–1997, Minya governorate, children <5 years of age of currently married women who were sick in 2 weeks before interview, n = 1579. SEP: One of risk factors.	**IO: Mother's education level** (none, primary, preparatory, secondary and higher, **mother's ever-employment for cash** (binary), **mother's ownership of assets** (none, one, two or more), **father's years education** (none, 1–11, 12+). **HH: Number of assets/durables owned:**(none, low [1 asset/0–2 durables], medium [≤1 asset, 3+ durables], high [2+ assets]. (Assets include 6 items of means of transportation, land and building ownership; Durables are 10 household assets such as TV, fridge etc.)	If reported symptoms of illness (with diarrhoea, fever, cough or rash): **Utilized private treatment (private doctor).** Level: 18.8%.
4	Fadel, et al. 2007[29]	Pros-pective cohort	257 healthy infants (0–9 months) followed for 12 months (October 1999–October 2000) in Assiut university primary health center catchment area, n = 631 diarrhoeal episodes. SEP: One of risk factors.	**IO: Mother's education, father's education** (illiterate, literate/primary, preparatory, secondary, university/higher), **mother's employment status** (binary). **HH: TV ownership** (binary), **Radio ownership** (binary).	If reported symptoms of diarrhoea in preceding 2 weeks: **Sought any treatment** (caretakers of children sought medical care outside the home). Level: 53.1% of diarrhoeal episodes (335 of 631). **Utilized private treatment** (medical care not under government control). Level: 37.3% of diarrhoeal episodes for which care was sought (210 of 335).
5	Gwatkin,et al, 2007[13]	Cross-sectional	EDHS 1995, 2000 (children under 5 years), n immunization: 1995 = 2085, 2000 = 2170; treatment for ARI: 1995 = 2479, 2000 = 1032; treatment for fever: 1995 = 4295, 2000 = 1923; treatment for diarrhoea: 1995 = 1701, 2000 = 771. SEP: Main determinant.	**HH: DHS Wealth index quintile**	**Coverage of immunisations:** BCG: 1995: 94.7%, 2000: 99.3%. Measles: 1995: 89.2%, 2000: 96.9%. DPT(3 doses): 1995: 83.0%, 2000: 94.0%. Hepatitis B: 1995: 57.0%, 2000 not reported. Full basic: 1995: 79.1%, 2000: 92.2%. No basic (not received full basic): 1995: 2.5%, 2000: 0.2%. **Sought any medical treatment.** Fever: 1995: 47.6%, 2000: 35.1%. ARI: 1995: 61.7%, 2000: 66.0%. Diarrhoea: 1995: 47.5%, 2000: 46.3%. **Utilized private treatment.** Fever: 1995: 34.8%, 2000: 21.7%. ARI: 1995: 43.5%, 2000: 41.5%. Diarrhoea: 1995: 33.2%, 2000: 30.0%. **Utilized public treatment.** Fever: 1995:12.0%, 2000: 12.6%. ARI: 1995: 17.1%, 2000: 23.1%. Diarrhoea: 1995: 13.7%, 2000: 15.6%.

EDHS – Egypt Demographic and Health Survey EGP – Egyptian pound BCG – Bacillus Calmette–Guérin (TB) DPT- Diphtheria, pertussis (whooping cough) and tetanus. Measures of SEP: **IO:** Individual – other (spouse, mother, etc), **HH:** Household-level.

### Maternal health-seeking behaviours

Three of the six studies analysing maternal health-seeking behaviours were based on the nationally representative population-level Demographic and Health surveys (DHS) collected between 1995 and 2008.[13]–[15] The remaining three studies analysed small samples of (<250) women in specific geographic locations; the city of Alexandria,[16] six villages in Beni Sueif governorate [17] and a Giza village.[18] All six studies were based on cross-sectional data.

**Figure 2:**
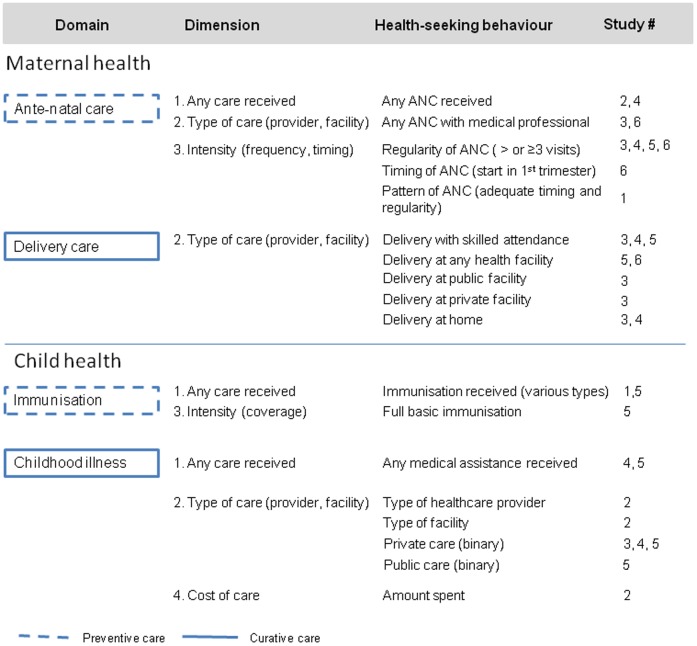
Maternal and child health-seeking behaviours analysed in included studies.

Four studies analysed whether women received any ante-natal care (ANC) or ANC from a medical professional during pregnancy (Figure 2, Table 2). The proportion of women receiving any ANC in the samples used by the studies ranged from 33.8% to 71.4% for any ANC and 42.5% to 73.2% for any ANC with a medical professional. Four studies examined ANC regularity (number of ANC visits during pregnancy) and one study each looked at the timing of the first ANC visit and at the pattern of ANC use (combining regularity and timing into one indicator). In regard to delivery care, four studies assessed various characteristics of the health provider (skilled or traditional birth attendant) or facility (any, public, private, home) where women sought care.

Four studies of maternal health-seeking behaviour used women's individual-level SEP measures; education or literacy appeared in four studies and employment-related SEP measurement reflected in occupation status and cash income earnings were used by one study each. Two studies evaluated individual-level SEP measures (education and occupation) of the spouse. All studies that analysed DHS surveys relied partly or solely on quintiles of the wealth index, a household-level measure.[19] Other household-level measures of SEP included family socio-economic status (SES) based on a score suggested in 1983 by Fahmy and Sherbini [20] and binary variables indicating ownership of various assets (land, house, cattle), access to potable water, and monthly meat consumption. One study examined the association between health-seeking behaviour and community-level economic prosperity, which was constructed as mean of the DHS household-level wealth scores within each survey primary sampling unit (PSU). Two of the six studies showed the absolute levels of health-seeking behaviours under investigation for each SEP category. To assess the existence and magnitude of the association between SEP and maternal health-seeking behaviours, four studies used odds ratio measure of effect and two studies presented the value of the concentration index, both relative effect measures.

#### Ante-natal care

The two studies which analysed more than one DHS survey both reported only a crude measure of association between SEP and health-seeking behaviours (Gwatkin 2007, Khadr 2009), shown in Table 4. However, their use of concentration index, a method which quantifies the extent of a SEP gradient, allowed for comparison of time trends. Concentration index is formally expressed as twice the area under the Lorenz curve of inequality, showing the cumulative distribution of an outcome according to cumulative distribution of wealth. Concentration index values range between -1, indicating absolute inequality of the outcome concentrated among the poorest and +1, showing absolute inequality concentrated among the wealthiest, with 0 signifying perfect equality. The definitions of health-seeking behaviours reported were similar (except for a difference in receiving ‘regular’ ANC, where definition varied between Gwatkin ≥3 and Khadr ≥4 visits). Both studies showed that women from lower wealth quintiles were significantly less likely to have received any ANC and regular ANC than women from richer wealth quintiles. Concentration index values for 1995 and 2000 differed between the two studies for the four indicators they both evaluated. Wealth score and quintile became available to users of the DHS starting with the 2005 survey, and authors calculated their own wealth index for the two prior surveys, possibly using different component variables, which may be the main reason for this difference. For both indicators of ante-natal care, the concentration index value decreased between the 1995 and 2005 surveys, but remained higher for regular ANC than for any ANC use. Khadr found that while the concentration index value for these two ANC behaviours based on education level remained significant and concentrated among women with higher SEP; the values were closer to equality than the concentration index values based on wealth quintile.

**Table 4:** Summary of results from included maternal health-seeking studies.

#	Author, Year	Statistical method, type of analysis, effect estimate	Results and confounders (if applicable)
1	Abdel Houssein, 1997[16]	**Crude analysis:** Not reported. **Adjusted analysis:** Logistic regression. **Effect estimate:** Odds ratio.	**Pattern of attendance of ANC clinics:** Education (number of categories not reported): OR 1.42 (0.64–3.13), p = 0.387. SEP (ref category not specified) OR 1.38 (0.75–2.56), p = 0.302. **Confounders:** age group (<20, 21–29, 30+), parity (0, 1–4, 5+), knowledge about adequate ANC use, level of risk, and health belief model score.
2	Yassin et al., 2003[17]	**Crude analysis:** Logistic regression. **Effect estimate:** Odds ratio.	**Any ANC:** Maternal literacy (ref: literate) OR 3.3 (1.6–6.7), p<0.001. Maternal occupation (ref: unskilled worker) OR 0.8 (0.3–1.8), p>0.05. Paternal literacy (ref: literate) OR 3.1 (1.4–6.9), p<0.01. Paternal occupation (ref: unskilled worker) OR 1.8 (0.7–4.5), p>0.05. Land ownership (ref: yes) OR 3.6 (1.4–9.3), p<0.01. House ownership (ref: yes) OR 1.0 (0.3–3.5), p>0.05. Cattle ownership (ref: yes) OR 0.5 (0.3–1.1), p>0.05. Access to safe water OR 1.1 (0.6–2.2), p>0.05. Meat consumption (ref: <4 times/month) OR 0.9 (0.4–2.1), p>0.05.
3	Gwatkin et al., 2007[13]	**Crude analysis:** Concentration index (CoI)*. **Effect estimate:** Concentration index value.	**Any ANC by a medically trained person:** Women from households in lower quintiles were less likely to receive but CoI decreased from 0.2703 (p<0.05) in 1995 to 0.2028 (p<0.05) in 2000. **Multiple (3+) ANC visits:** Women from households in lower quintiles were less likely to receive multiple ANC visits, but CoI decreased from 0.3580 (p<0.05) in 1995 to 0.2614 (p<0.05) in 2000. **Delivery with skilled attendance:** Women from households in lower quintiles were less likely to deliver with skilled medical assistance, but CoI decreased from 0.2911 (p<0.05) in 1995 to 0.2189 (p<0.05) in 2000. **Delivery location:** Public or Private facility: Women from households in lower quintiles were less likely to deliver in private facilities (CoI1995: 0.4379, p<0.05; CoI2000: 0.3422, p<0.05) and in public facilities (CoI1995: 0.2472, p<0.05; CoI2000: 0.1708, p<0.05), both indexes decreased between 1995 and 2000. **Delivery location: Home:** Women from households in lower quintiles were more likely to deliver at home and gradient increased over time (CoI1995: −0.1630, p<0.05; CoI2000: −0.2457, p<0.05).
4	Khadr, 2009[14]	**Crude analysis:** Concentration index (CoI). **Effect estimate:** Concentration index value.	**Any ANC received:** Education: CoI value has remained the same (0.41) between 1995 and 2005 (p<0.001). Wealth: Decreased inequality from 0.50 (1995) to 0.45 (2005) but significant (p<0.001). **Regular ANC received:** Education: Decreased inequality from 0.49 (1995) to 0.39 (2005) but significant (p<0.001). Wealth: Decrease in inequality from 0.60 (1995) to 0.49 (2005) but significant (p<0.001). **Delivery with skilled attendance:** Education: Decrease in inequality from 0.41 (1995) to 0.37 (2005) but significant (p<0.001). Wealth Decrease in inequality from 0.55 (1995) to 0.47 (2005) but significant (p<0.001). **Home delivery:** Education: Decrease in inequality from −0.42 (1995) to −0.35 (2005) but significant (p<0.001). Wealth: Decrease in inequality from −0.53 (1995) to −0.47 (2005) but significant (p<0.001).
5	Chiang et al., 2012[18]	**Crude analysis:** Logistic regression. **Effect estimate:** Odds ratio.	**Regular ANC:** Maternal education (ref: no primary education) OR 5.59 (2.98–10.47), p<0.01. Cash income (ref: no) OR 0.52 (0.25–1.09), p>0.05. Husband education (ref: ≤primary) OR 2.31 (1.26–4.23), p<0.01. **Delivery with skilled medical professional:** Maternal education (ref: no primary education) OR 2.98 (1.36–6.54), p<0.01. Cash income (ref: no) OR 0.79 (0.34–1.85), p>0.05. Husband education (ref: ≤primary) OR 2.38 (1.05–5.36), p<0.05. **Delivery in a health facility:** Maternal education (ref: no primary education) OR 5.42 (2.78–10.57), p<0.01. Cash income (ref: no) OR 0.51 (0.25–1.04), p>0.05. Husband education (ref: ≤primary) OR 2.82 (1.46–5.42), p<0.01.
6	Stephenson et al., 2012[15]	**Crude analysis:** Not reported. **Adjusted analysis:** Multi-level logistic regression. **Effect estimate:** Odds ratio.	**Any ANC received:** OR 1.0 (1.0–1.0), p>0.05. **Regular ANC received:** OR 1.0 (1.0–1.0), p>0.05. **First ANC in 1^st^ trimester:** OR 1.0 (1.0–1.0), p>0.05. Delivery in a health facility: OR 1.0 (1.0–1.0), p>0.05. **Confounders:** Woman's age, marriage duration, partner age difference, death of a child, number of living children, sex ratio of children, education (woman and partner), employment status (woman and partner), household wealth, reproductive health knowledge, media exposure, violence justification, and decision-making autonomy.

* Only values of standard error (SE) were provided by this study. Binary significance levels (<0.05, >0.05) were calculated by multiplying the SE by 1.96 to obtain the upper and lower confidence intervals.

In order to approximate the level of community affluence, Stevenson et al (2012) constructed a community level wealth index based on the household wealth index of respondents in the same primary sampling units. This study concluded that community-level economic prosperity was not associated with any ANC, regular ANC or timely ANC (first visit in first trimester of pregnancy) use. Receiving regular ANC in a Giza village was associated with primary or higher education level - both woman's as well as her husband's, but not with woman's cash income, in crude analysis (Chiang 2012). Crude analysis of a sample from villages in Beni Sueif showed a significant positive association between maternal and paternal literacy (individual-level) and land ownership (household-level) with the likelihood of receiving any ANC (Yassin 2003). A study in Alexandria reported that neither education nor family SES score were significantly associated with adequate pattern of ANC clinic attendance among women attending an ANC clinic, when adjusted for woman's age group, parity, knowledge of ANC use, level of medical risk, and health belief model score (Abdel Houssein 1997).

#### Delivery care

The concentration index values for obtaining skilled delivery care appeared to have decreased between 1995 and 2005, yet the values remained significant, and higher based on wealth quintile than on education level (Gwatkin 2007, Khadr 2009). Gwatkin and colleagues reported that women from lower household wealth quintiles were significantly less likely to deliver in private facilities and in public facilities, compared to women from higher quintile households, in both 1995 and 2000, but the concentration index value had decreased over this timeframe. Both Gwatkin and Khadr concluded that women from lower wealth quintiles were significantly more likely to deliver at home in all surveys analysed. However, whereas Gwatkin reported that the concentration index value increased between 1995 and 2000, Khadr noted a decrease between 1995 and 2005. Khadr's analysis showed that the concentration index value for home delivery was smaller when SEP was measured by education level rather than household wealth quintile, but remained significant, favouring women with higher SEP. Using the 2008 DHS, Stephenson et al reported no significant difference, in adjusted analysis, in the odds of delivery in any health facility compared to at home based on community-level wealth. Lastly, crude analysis of health-seeking behaviour in a Giza village revealed that higher level woman's and husband's education were significantly positively associated with the odds of delivery with a skilled medical professional and delivery in any health facility. However, woman's cash income was not associated with either delivery-related health-seeking behaviour (Chiang 2012).

### Child health-seeking behaviours

Five studies analysing SEP determinants of health-seeking behaviour for children were identified; two included assessment of immunisation coverage and four examined aspects of curative health-seeking for common childhood illnesses (Figure 2, Table 3). Two studies analysed nationally-representative samples; one by following up on National Immunisation Days (Reichler 1998) and the other examined two rounds of EDHS (Gwatkin 2007). Two papers reported findings from samples in Minia governorate based on the Two Governorates Linkages Survey (Yount 2003 and Yount 2004), and the remaining study assessed health-seeking behaviours for infants in a primary health centre catchment area in Assiut (Fadel 2007). Although this study included prospective cohort data collection method, all findings were based on cross-sectional analyses.

Between the two studies examining receipt of immunisations, socio-economic determinants of both disease-specific coverage (BCG, measles, DPT, Hepatitis B, polio), as well as overall immunisation status based on national guidelines (full basic immunisation, no basic immunisation) were assessed. In regard to curative care, health-seeking behaviours following reported childhood illnesses (any illness, diarrhoea, acute respiratory infection-ARI, or fever) were examined, specifically whether any medical care was sought, the type of medical provider and health facility approached, and cost incurred. Three of the five studies used measures of parental SEP, namely maternal and paternal education level and employment status, and maternal asset ownership. All three studies using individual-level variables also used household-level measures of SEP, the remaining two studies analysed solely household-level measures. The household-level SEP measures included DHS wealth quintiles, binary TV and radio ownership, and major asset ownership. Four of the five studies showed the absolute levels of health-seeking behaviours under investigation for each SEP category. The assessment of the association between SEP and maternal health-seeking behaviours was conducted using relative effect measures in four studies (three used odds ratios and one concentration index value) and absolute measure in one study (risk difference).

#### Immunisation coverage

Both studies examining the association between SEP and receipt of immunisations presented only crude effect estimates (Table 5). In their analysis of polio vaccine coverage during the 1995 National Immunisation Days, Reichler et al concluded that children living in households with a radio or a TV were significantly more likely to have received two oral polio virus vaccine doses than one or zero doses. Using concentration index based on household wealth quintile, Gwatkin et al reported that children from lower quintile households were less likely to be immunised against BCG, measles, and DPT (3 doses) than those from higher quintiles, although the concentration index value had decreased between 1995 and 2000. The concentration index value for Hepatitis B immunisation was only available for 1995 and showed the highest level (higher coverage among children from wealthier quintile households) among the various vaccines. Children from lowest wealth quintile households were significantly more likely to have received no basic immunisation coverage, and the concentration index value declined only marginally in the five-year period between surveys.

**Table 5:** Summary of results from included child health-seeking studies.

#	Author, Year	Statistical method, type of analysis, effect estimate	Results and confounders (if applicable)
1	Reichler et al., 1998[26]	**Crude analysis:** T-test. **Effect estimate:** Risk difference.	Children in households with a radio were more likely to have received 2 doses (77%) than one (69%) or no doses of OPV (72%), p<0.01. Children in households with a TV were more likely to have received 2 doses (94%) than one (86%) or no doses of OPV (84%), p<0.001.
2	Yount, 2003[27]	**Crude analysis:** Not reported. **Adjusted analysis:** Logistic regression. **Effect estimate:** Odds ratio.	**Type of provider**: Household assets (ref: none) Any: Pharmacist OR 1.78 (p>0.05), Doctor OR 2.46 (p>0.05). Maternal education (ref: none) Any: Pharmacist OR 2.10 (p>0.05), Doctor OR 0.75 (p>0.05). **Location of care**: Household assets (ref: none) Any: Public OR 3.65 (p>0.05), Pharmacy OR 2.01 (p>0.05), Private OR 2.20 (p>0.05). Maternal education (ref: none) Any: Public OR 0.66 (p>0.05), Pharmacy OR 2.09 (p>0.05), Private OR 0.72 (p>0.05). **Cost of care** (categorical variable, 3 levels): Household assets (ref: none) Any: OR 0.76 (p>0.05). Maternal education: not reported. **Confounders**: child gender and age; severity, cause and duration of diarrhoea; presence and gender of siblings; and family members' involvement in decision-making.
3	Yount, 2004[28]	**Crude analysis:** Not reported. **Adjusted analysis:** Logistic regression. **Effect estimate:** Odds ratio.	**Odds of utilizing private treatment:** Father's education (ref: 0 years): 1–11 years OR 0.84 (p>0.05); 12+ years OR 1.10 (p>0.05). Maternal ever-employment (ref: no): Yes: OR 0.79 (p>0.05). Maternal education (ref: none): Primary/preparatory: OR 0.81 (p>0.05), secondary or more: OR 0.92 (p>0.05). Maternal assets (ref: none): One OR 0.74(p<0.05), Two or more: OR 0.79 (p>0.05). Household assets/durables (ref: none): Low: OR 1.84 (p<0.01), Medium: OR 2.55 (p<0.001), High: OR 3.62 (p<0.001). **Confounders**: child gender and age; severity, cause and duration of illness; presence and gender of siblings; religion; urban/rural residence; maternal social resources and social constraints; and proximity of health services.
4	Fadel, et al. 2007[29]	**Crude analysis:** X^2^ test. **Adjusted analysis:** Logistic regression. **Effect estimate:** Odds ratio.	**Crude analysis - Utilized treatment:** Mother's education p = 0.285, Father's education p = 0.339, Mother's employment status p = 0.486, TV ownership p = 0.042 (diarrhoeal episodes where households have TV more likely to utilize care), Radio ownership p = 0.196. **Utilized private providers, if utilized treatment:** Mother's education p<0.001 (higher levels of education are more likely to utilize private providers), Father's education p = 0.020 (higher levels of education are more likely to utilize private providers), Mother's employment status p<0.001 (working mothers are more likely to utilize private providers), TV ownership, Radio ownership: not reported. **Adjusted analysis - Utilized treatment:** Mother's education, Father's education, Mother's employment status, Radio ownership: not reported. TV ownership (ref: not clear) OR 0.63, p = 0.418. **Confounders**: Perception of severe attack, vomiting, bloody stool, fever, duration of episode, age of infant, frequency of stool, presence of cough, associated any symptoms with diarrhoea, watery stool, history of child death, presence of preschool children, using self-prescribed drugs. **Utilized public versus private providers:** Mother's education (ref: illiterate): reads/writes/primary: OR 0.04 (p<0.001), preparatory: OR 0.02 (p<0.001), secondary: OR 0.04 (p<0.01), university/higher: OR 0.07 (p<0.01). Father's education (ref: illiterate): reads/writes/primary: OR 2.04 (p = 0.212), preparatory: OR 1.27 (p = 0.726), secondary: OR 0.82 (p = 0.785), university/higher: OR 1.93 (p = 0.209). Mother's employment status (ref: not working): working: OR 0.61 (p = 0.278). TV ownership, radio ownership: not reported. **Confounders**: age and sex of infant, mother's age and mother's perception of diarrhoeal episode severity.
5	Gwatkin, et al, 2007[13]	**Crude analysis:** Concentration index (CoI)*. **Effect estimate:** Concentration index value.	**Coverage of immunisation:** BCG: Children from lower quintiles less likely covered, CoI decreased from 0.0317 (p<0.05) in 1995 to 0.004 (p<0.05) in 2000. Measles: Children from lower quintiles less likely covered, CoI decreased from 0.058 (p<0.05) in 1995 to 0.009 (p<0.05) in 2000. DPT: Children from lower quintiles less likely covered, CoI decreased from 0.0691 (p<0.05) in 1995 to 0.0022 (p>0.05) in 2000. HepB: Children from lower quintiles less likely covered, 0.0984 (p<0.05) in 1995, no 2000 estimate. Full basic: Children from lower quintiles less likely covered, CoI decreased from 0.0819 (p<0.05) in 1995 to 0.0052 (p>0.05) in 2000. No basic: Children from lower quintiles more likely to have no coverage, CoI decreased from −0.7434 (p<0.05) in 1995 to −0.7212 (p>0.05) in 2000. **Sought any treatment**: Fever: Children from lower quintiles less likely to receive, CoI decreased from 0.1076 (p<0.05) in 1995 to 0.077 (p<0.05) in 2000. ARI: Children from lower quintiles less likely to receive, CoI decreased from 0.0965 (p<0.05) in 1995 to 0.0897 (p<0.05) in 2000. Diarrhoea: Children from lower quintiles less likely to receive, CoI increased from 0.0357 (p<0.05) in 1995 to 0.0688 (p<0.05) in 2000. **Utilized private provider:** Fever: Children from lower quintiles less likely to receive, CoI increased from 0.1609 (p<0.05) in 1995 to 0.1928 (p<0.05) in 2000. ARI: Children from lower quintiles less likely to receive, CoI increased from 0.1543 (p<0.05) in 1995 to 0.1932 (p<0.05) in 2000. Diarrhoea: Children from lower quintiles less likely to receive, CoI increased from 0.1095 (p<0.05) in 1995 to 0.1713 (p<0.05) in 2000. **Utilized public treatment**: Fever: Children from lower quintiles more likely to receive, CoI increased from −0.0411 (p>0.05) in 1995 to −0.1240 (p<0.05) in 2000. ARI: Children from lower quintiles more likely to receive, CoI increased from −0.0469 (p<0.05) in 1995 to −0.0892 (p<0.05) in 2000. Diarrhoea: Children from lower quintiles more likely to receive, CoI decreased from −0.1446 (p<0.05) in 1995 to −0.1180 (p<0.05) in 2000.

* Only values of standard error (SE) were provided by this study. Binary significance levels (<0.05, >0.05) were calculated by multiplying the SE by 1.96 to obtain the upper and lower confidence intervals.

#### Curative care

In terms of the first dimension of curative health-seeking, seeking advice from a medical provider, Gwatkin's crude analysis showed that children from lower quintiles were less likely to receive care with symptoms of fever, acute respiratory infection (ARI) and diarrhoea compared to children from higher quintile households. However, whereas the concentration index value decreased between 1995 and 2000 for symptoms of fever and ARI, these values increased with respect to diarrhoea. The highest concentration index value in 2000 for this dimension was seen in health-seeking for symptoms of ARI. In a small sample of infants in Assiut, Fadel examined the association between SEP and seeking medical advice following symptoms of diarrhoea. In crude analysis, only household TV ownership was marginally positively associated with seeking care, but ceased to be significant in adjusted analysis.

Four studies examined the association between SEP and type of care chosen for childhood illness among the subsamples of children for whom any medical care was sought. In the nationally-representative DHS surveys, Gwatkin reported results of a crude association, finding that children from lower quintile households were less likely to have received care from a private provider. Whereas the concentration index value was smaller for symptoms of diarrhoea compared to ARI or fever in both surveys, the value had increased for all three illnesses between 1995 and 2000. Among infants in Assiut for whom medical care was sought for diarrhoea, utilisation of private care was associated with higher levels of both mother's and father's education, as well as mother's employment status in crude analysis (Fadel 2007). Among these three SEP indicators, only mother's education remained significantly positively associated with private care utilisation in adjusted analysis. In a sample of children taken for care for diarrhoea, fever, cough or rash in Minia, adjusted odds of seeking a private doctor were significantly higher for those with higher number of maternal as well as household assets, but not associated with maternal or paternal education or maternal ever-employment (Yount 2004). Analysing a smaller sample of children with diarrhoea in Minia, Yount (2003) found that neither the type of medical provider (doctor, pharmacist, other, lay) nor the type of facility (public, private, pharmacy) sought was associated with maternal education or household assets in adjusted analysis. This study also found no association, after adjustment for various confounders, between household asset ownership and cost of care incurred.

## Discussion

Studies using nationally representative datasets found evidence of an association between SEP and maternal health-seeking behaviours. Women living in lower wealth quintile households were less likely than those from wealthier households to receive any and regular ANC, to deliver with skilled attendance and in health facilities. The magnitude of this association appeared smaller when women's SEP position was measured by education level compared to household wealth. However, no association was found between community-level affluence and maternal health-seeking behaviours. These differences in the existence and extent of SEP gradients raise the question whether material/financial resources may be a more important determinant of utilisation (potentially via direct and indirect costs of care) than women's knowledge of the need for care or its availability. On the other hand, the only adjusted analysis of a small sample found that women's education or household-level SES score did not predict the pattern of ANC attendance among women attending ANC clinics, potentially suggesting that once women access ANC care, determinants other than SEP contribute to the intensity of care they receive.

Children from wealthier households were significantly more likely to have been immunised, for separate illnesses and for a combination of basic immunisations, compared to children from poorer households. Analysis of time trends revealed that this gradient had decreased between 1995 and 2000, potentially as a result of high overall immunisation coverage. Crude analysis of a nationally representative sample showed that the gradient in care-seeking for symptoms of childhood illnesses favoured children from higher wealth quintile households, but while remaining significant, decreased over time. A significant and increasing inequality in private provider use based on household wealth was identified for all three childhood illnesses examined. This may be a result of the fact that the cost of private care is higher than of public care.[21], [22] However, in smaller samples from Upper Egypt, conflicting results about the existence of an association between SEP and utilisation of any curative care and private provider selection for child illnesses were reported. This may be due to real differences between the populations from which samples were drawn, or be partially or fully explained by the difference in analysis methods (crude versus adjusted) or definition of illness (diarrhoea and any illness). The evidence on existence of an association between SEP and the cost of care-seeking was limited to one study (not adjusted for provider type) of a small sample, and therefore carried limited generalizability.

### Limitations of available evidence

We considered selection bias, information bias (related to exposure or outcome) and analysis methods in assessing data quality of each study (Table 6).

**Table 6:** Risk of bias in included studies.

	Health-seeking behaviour	Maternal						Child				
Risk of bias category	Study	Abdel Houssein, 1997	Yassin, 2003	Gwatkin, 2007	Khadr, 2009	Chiang 2012	Stephenson, 2012	Reichler, 1998	Yount, 2003	Yount, 2004	Fadel,2007	Gwatkin, 2007
**Selection**	Representativeness (consecutive or obviously representative)	−	?	+	+	?	+	+	?	?	?	+
	Missing data or loss to follow up minimal	?	?	+	+	?	+	?	?	?	?	+
**Exposure (SEP)**	Clear definition of exposure	−	+	?	+	+	?	+	+	+	+	?
	Ascertainment of exposure	?	+	+	+	+	+	+	+	+	?	+
**Outcome (HSB)**	Clear definition of outcome	−	+	+	+	+	+	+	?	?	+	+
	Ascertainment of outcome	−	+	?	?	+	+	?	+	+	+	?
**Analysis**	Statistical test used to analyze the data is clearly described* and appropriate	+	+	+	+	+	?	+	+	+	+	+
	Minimal adjustment for age (maternal, child), parity (maternal) and gender (child)	+	−	−	−	−	+	−	+	+	+	−
	Correctly adjusting for several indicators of SEP	−	NA	NA	NA	NA	−	NA	−	−	−	NA

SEP – socio-economic position, HSB – health-seeking behaviour.

* Presenting a test of statistical significance was one of the inclusion criteria.

NA – not applicable (no adjusted analysis was conducted).

Key: + Low risk of bias, ? Potential/unclear risk of bias, - High risk of bias.

Five studies used nationally representative survey data. Whereas the remaining smaller surveys considered selection bias (by including, for example, all women attending ANC, all mothers living in villages, or utilising simple random sampling in a community), they often neglected to describe the specifics of the population their samples represent. It was unclear whether sample sizes were calculated based on sufficient power to detect gradients in health-seeking behaviours, or whether previously collected data was used in analysis of health-seeking. Therefore, the findings of several such studies have limited generalizability not only on the general population level, but also for the understanding of local-level determinants of health-seeking. All included studies relied on cross-sectional analysis, and no analysis of individual-level time patterns of health-seeking behaviours was presented.

Two of the included studies specifically analysed socio-economic determinants of health-seeking behaviours. Seven studies attempted to identify determinants of health-seeking behaviours and one paper included SEP as a confounder in analysis of gender. Studies using separate variables, such as education level or binary asset ownership described the definition and categorisation of exposure categories better than those using component variables (SES score, household wealth quintile). Potential bias may stem from data collection methods – studies failed to report on and examine which member of the household reported on the various indicators of SEP (own, other members' or household-level) and whether this person could do so reliably.

Limitations in the definitions of health-seeking behaviours in included studies relate mainly to type of provider or facility. The method of categorising health providers into skilled or unskilled and health facilities into public or private was not always made explicit and may have relied of respondents' self-report. This concern is particularly pertinent to the use of pharmacies without previous medical consultation, a common practice in Egypt. The existence and extent of an SEP gradient by provider type may change as a result of the inclusion and categorization of pharmacies. Misclassification may have occurred in regard to type of provider, because many public sector medical professionals also practice privately. In addition, respondents may not know, correctly recall, or be willing to report the level of medical qualification of the provider used. The recall period used by studies for reporting of health-seeking behaviours seemed appropriate – longer for maternal care where information surrounding a birth is more likely to be remembered, and shorter (two weeks preceding survey or prospective data collection) for events related to child health. Child curative care relies heavily on the perception and reporting of child ill health. However, only Yount 2004 and Fadel 2007 used perceived severity of illness in their adjusted analyses of health-seeking determinants. On the conceptual level, the use of such an approach also challenges the underlying assumption that all perceived childhood illness symptoms ought to be attended to by a health provider.

Several important gaps in the body of evidence are present. In regard to maternal health-seeking, no study analysed SEP determinants of the type of facility (public or private) chosen by users of ante-natal care. Further, no study modelled the intensity of ANC care as a continuous variable, which would enable detailed analysis of the dose-response effects of SEP. No analysis of post-natal care, a crucial component of the maternal health package, was identified. Neither the determinants of facility level (health centre, hospital) nor of a combination of provider qualification and facility characteristics were examined for delivery care health-seeking. Lastly, expenditures related to maternal health-seeking and their association with SEP were not examined by any study. In comparison with maternal care, available evidence for child health-seeking included more dimensions of behaviours related to curative, but less for preventive care. No studies analysed types of providers and facilities approached or costs encountered in obtaining childhood immunisations. No evidence examining gradients in intensity of care (number of visits made and types of providers approached, if more than one) received for childhood illnesses was identified. Lastly, the type of symptoms for which curative health-seeking was analysed is limited to three; no evidence examining other important issues, such as childhood injuries or mental health was identified.

The use of a statistical test to assess the existence and extent of SEP gradients was an inclusion criterion in this review. However, included studies suffered from several other limitations in their analyses. Six studies only presented results of crude analyses and this was a major limitation of the usefulness of studies that employed nationally-representative data. The usefulness of analyses to policy is also limited by the number of following dimensions of health-seeking behaviour assessed in any given study. A gradient in intensity of care (dimension 3) may not only be a result of inequalities experienced at that point, but also a consequence of accumulated inequalities in seeking care (dimension 1) and the type of provider used (dimension 2). For example, in the only analysis of cost of care identified by this review (Yount, 2003), adjusted analysis did not include provider type and cannot therefore elucidate the SEP gradient in expenditures based on provider choice. Further, all five studies presenting adjusted analyses used various SEP indicators in one adjusted model, thereby controlling for (eliminating) the pathways of association from one SEP indicator through those remaining.

### Strengths and limitations of systematic review

In addition to limitations of the studies included in this review and the gaps in the body of evidence, limitations of this systematic review were also assessed. The search strategy targeted global and regional databases, but only English sources were searched. Complete retrieval of studies identified for full-text review was achieved. Health-seeking behaviour is not a new concept, although it is a recent term, and care was taken to identify and use as many synonyms and components as possible in the text and MeSH terms used. Likewise, a wide range of potential SEP indicators was compiled and used in searches. However, the search strategy may have missed studies which used socio-economic indicators as confounders, but did not list them in the title, abstract or keywords. One author was responsible for study selection and data extraction. Most studies looked at a range of factors associated with health-seeking behaviours, not solely SEP. Therefore, while no formal assessment of publication bias was conducted, it is possible that studies which failed to find an association between health-seeking behaviour and SEP did not report the estimates. Such studies may also not have been submitted or accepted for publication. Despite the fact that only quantitative studies assessing the existence and extent of SEP gradient were included in the review, it was not possible to produce a summary measure of association due to the variability of definitions, effect estimates and analysis methods in individual studies.

## Conclusion

Following large improvements in coverage of maternal and child health interventions in Egypt, socio-economic inequalities, and in particular the rural concentration of poverty, have been identified as the primary determinants of remaining disparities.[23], [24] As one of the pathways in this association, health-seeking behaviours could contribute to such gradient in health outcomes. While the body of evidence presented in this study contains several limitations, we have attempted to synthesise the available evidence related to inequalities in dimensions of maternal and child health-seeking behaviour.

On the national level, socio-economically patterned inequalities in seeking any care for basic preventive and curative interventions in maternal and child health appear to have narrowed, potentially as a result of increased overall coverage. However, the extent of this gradient seems larger measured by wealth compared to education, and further exploration to determine whether and how knowledge and affordability of care drive these inequalities is necessary. On the other hand, the limited evidence of gradients in intensity of preventive care in maternal, and provider type in child curative care, showed that inequalities may have widened. In studies of more geographically and socially homogenous samples, fewer gradients were identified, signifying that in areas with comparable health service supply, future research will need to examine determinants of health-seeking beyond the traditionally used SEP indicators.

Overall, although ten studies examining socio-economic inequalities in health-seeking behaviours were identified, the body of research contains numerous gaps and the quality of available evidence is insufficient to draw a conclusive summary of the extent of gradients in Egypt. Future research needs to address gaps in the assessment of the various dimensions of maternal and child health-seeking behaviours, while carefully defining constructs underlying SEP indicators and correctly modelling this association in statistical analyses. This understanding will be crucial in designing and prioritising interventions to equitably improve maternal and child health outcomes in Egypt.[25]

## Supporting Information

Table S1 **PRISMA guidelines checklist.**
(DOCX)
